# Modelling the Effects of Treatment Failure on the Minor Outbreak Duration for Carrier-Related Infectious Disease

**DOI:** 10.3390/epidemiologia7030058

**Published:** 2026-04-22

**Authors:** Pichaya Voottipruex, Nichaphat Patanarapeelert, Klot Patanarapeelert

**Affiliations:** 1 Department of Mathematics, Faculty of Applied Science, King Mongkut’s University of Technology North Bangkok, Bangkok 10800, Thailand; pichayavoo@gmail.com (P.V.); nichaphat.p@sci.kmutnb.ac.th (N.P.); 2 Department of Mathematics, Faculty of Science, Silpakorn Universtiy, Nakhon Pathom 73000, Thailand

**Keywords:** treatment failure, carriers, minor outbreak, branching process, extinction threshold

## Abstract

**Background:** The complex interplay between treatment interventions and asymptomatic carriers and its effect on the epidemic duration of an infectious disease is not fully understood. **Methods:** Here, we used Galton-Watson branching process and generating function technique to estimate the density functions of minor outbreak duration. Simulations were used to calculate the central tendency of outbreak duration and address how changing levels of treatment failure affect this estimated duration. **Results:** *Streptococcus pyogenes* infection was used as a case study. Given the existence of the threshold, the change in mean duration as the probability of treatment failure increases is shown to be similar to the pattern driven by the basic reproduction number. In a supercritical regime, the mean duration tends to decrease as the probability of treatment failure increases. The distribution changes in tail behavior, from heavy- to light-tailed, if a large fraction of long extinction times develops to a major outbreak. **Conclusions:** Treatment failure elevates the probability of secondary transmissions by prolonging the overall infectious period, resulting in an extended the outbreak duration. The threshold of treatment failure identifies the maximum tolerable error for medical intervention. An unusually long period implies a critical early warning signal of a potential major outbreak that was successfully contained.

## 1. Introduction

Treatment failure for infectious diseases usually implies not achieving the target therapeutic outcome, which includes non-response to treatment and patient worsening [1]. Its definition is not well-defined, depending on the specific pathogen, factors, and mechanisms. Antimicrobial resistance (AMR) is an example of bacteriological failure, referring to microorganisms’ ability to resist the effects of antibiotics, which is influenced by factors such as human practices [2]. Patient non-adherence, inadequate medical regimens, and immune status are common host-related factors, particularly relevant in long-term treatments for diseases like HIV or Tuberculosis. Genetic mutations in viruses can enable them to resist antiviral drugs, as seen in cases of HIV and influenza [3,4]. In addition, clinical factors such as inappropriate drug selection and inadequate treatment duration interact with both pathogen and host factors. These clinical issues can be influenced by misdiagnosis [5,6], testing strategies [7], and the failure to identify drug resistance [8].

Not just an adverse impact on the individual level, treatment failure can lead to the spread of resistant strains, prolonging the epidemic period of the susceptible strain and increasing the burden on public health systems [9]. Thus, the study of treatment failure is crucial for medical advancement to improve protocols that foster treatment-based interventions and disease management. Nevertheless, measuring the effectiveness of treatment amid the presence of asymptomatic carriers may be difficult since clinical interventions are often symptom-based.

An asymptomatic carrier is generally defined as an infected person who does not show any signs or symptoms of the infection. The hidden threat is that such carriers may be able to transmit a pathogen without preventive measures or control. It has been recognized that these asymptomatic carriers contribute to the spread of diseases such as COVID-19 [10,11], Tuberculosis [12], influenza [13], Streptococcal pharyngitis [14], and pneumococcal disease [15]. Depending on the disease state, carriers can transmit a pathogen during the presymptomatic or post-illness period, and may become chronic, acting as an invisible reservoir for long-term transmission. Since carriers are difficult to identify in general practice, even perfect treatment of symptomatic cases may not be sufficient to reduce the potential risk of an epidemic. In the case of acute respiratory viruses, such as SARS-CoV-2 or influenza, a timely treatment strategy may efficiently reduce morbidity but may only partially break the chain of close-contact transmission within a household, as the index case usually presents with symptoms [16].

In contrast, treatment failure can fuel transmission by delaying the infectious period and may create carriers due to the unsuccessful in eliminating the disease-causing pathogen from the body. Streptococcal carriers can be classified as “true” or “apparent” failures [17]. True failure refers to the result of incomplete eradication, while apparent failure is characterized by no symptoms of pharyngitis and/or no immunologic response to the antigens. The latter case is usually chronic and may not respond to regular antibiotic therapy [18]. As a consequence, an association between respiratory viruses and Group A Streptococcus is possible to cause misdiagnosis, resulting in treatment failure [19]. Thus, successful treatment of symptomatic cases could indirectly benefit epidemic prevention since it reduces the number of potential carriers.

Mathematical modelling plays a prominent role in completing a picture of how diseases truly spread under an incomplete and potentially misleading measure of the overall disease burden driven by the interplay between asymptomatic carriers and treatment failure. Treatment is traditionally unnoticed, as it may be implicitly described as isolation or hospitalization or hidden within the recovery rate. However, it has been purposely included in the design of epidemic dynamic models to evaluate the effectiveness of various control strategies [20,21,22,23,24,25]. Among several studies, only a few papers jointly account for the influence of asymptomatic carriers. For large-scale epidemics, models used to analyze the impact of carriers in treatment interventions include, for example, a control model of Tuberculosis transmission [12] and a dynamic model of a Meningococcal Meningitis outbreak, which incorporates undiagnosed carriers and hospitalization [26]. For small population scales, carrier-related models aim to evaluate the effectiveness of antiviral treatment strategies in households [16], and hospitals [27]. One of the challenges of using such models is describing an infection at the point of elimination or extinction [28].

Large-scale outbreaks normally begin as locally small outbreaks, but vice versa is not true. Instead of spreading widely, the epidemic may fizzle out due to its random nature and a few initially infected individuals. The potential to escalate of a minor outbreak deserves public health attention as a critical early warning signal. Thus, studying minor outbreaks of emerging or re-emerging diseases is crucial for preparing for major pandemics [29,30]. Yet, general criteria that determine the threshold for an outbreak to either die out or become a major one do not exist. Apart from the critical community size, a study by [31] defines the outbreak threshold as the sufficient number of infected individuals to ensure that the chain of transmission can temporarily escape extinction. It has been shown that a minor outbreak can develop into a major outbreak if three basic measures—such as incidence rate, final size, and outbreak duration—reach predetermined thresholds [32].

The aim of this study is to analyze the impact of treatment failure on the duration of minor outbreaks using an epidemic dynamic model that captures the role of asymptomatic carriers. As mentioned earlier, treatment failure can disproportionately impact an outbreak’s duration, creating a persistent source of infection. How this intervention alters the duration of a minor outbreak is not yet known. In general, an epidemic’s duration can be used alongside other metrics to characterize whether the outbreak is minor or major. However, a pathogen may persist in a population for a long period with only a small number of cases. This usually occurs when the basic reproduction number ($R_{0}$) is close to unity [33].

Recently, it has been shown that treatment failure can lead to a stochastic parametric threshold, below which extinction is almost certain [34]. We question whether the change in the duration of a minor outbreak due to treatment failure resembles the basic reproduction number. Despite this, such a parametric threshold may exist only under specific constraints; otherwise, the system may remain in either a subcritical or supercritical process independent of treatment failure. Thus, we hypothesize that there may be a difference between two scenarios for the change in minor outbreak duration within a supercritical process.

It is clear that stochastic models are required to properly describe disease extinction. Here, we extend a previous epidemic model and employ the Galton-Watson branching process and the generating function technique to estimate the duration of a minor outbreak. In a finite stochastic epidemic process, disease extinction is inevitable. There has been interest in estimating the “time to extinction” after the quasi-stationary state [35,36,37,38,39,40]. In this study, however, we focus on the time to extinction for processes that die out early, which provides a definition of minor outbreak duration in a branching process. The mean and median of the duration are calculated using numerical simulations. To this end, we use parameter values that describe the transmission dynamics of *S. pyogenes* as a case study.

## 2. Materials and Methods

### 2.1. Model Description

We highlight an epidemiological model that comprises susceptible (S), carriers (C), symptomatic infected (I), and individuals currently under treatment (T). To increase flexibility, we extend the previously studied model [34] by including that the infected may evolve from carriers to symptomatic infection, and by differentiating the treatment failure related with carriers from other possible causes. The first assumption was made in previous studies [41,42,43], whereas the latter allows us to examine the specific influence of carriers in the context of overall treatment failure.

**Figure 1:**
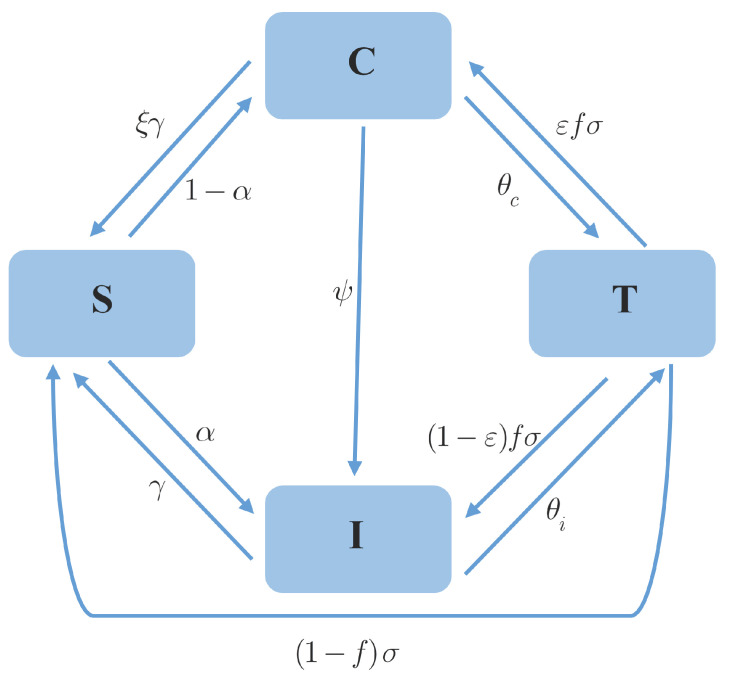
Compartmental epidemic model with carriers and treatment.

Figure 1 shows a flow diagram of states in the model. Once an infection occurs, we assume that susceptible individuals become symptomatically infected with a probability α, or enter the carriage state with a probability 1−α. The transmission rate of a symptomatic patient is given by β, while that of a carrier is given by ηβ, where η represents the relative transmission potential of carriers compared to symptomatic individuals. This is usually true when 0<η<1. We assume that symptomatic patients without treatment recover at a rate γ, while the recovery rate of carriers is usually lower by a factor ξ (0<ξ<1).

Although infected individuals undergoing treatment may exhibit transmission potential, their impact is low compared to the pre-treatment phase and untreated individuals. Factors such as transmission precautions, quarantine, restricted mobility, and a shortened infectious period support this remark especially during the early stages of an outbreak with a limited number of cases. For the sake of simplicity, we assume that the individuals in class *T* are unable to transmit the disease, as in the previous study [34]. Carriers and symptomatic infections enter state *T* at the rates $\theta_{c}$ and $\theta_{i}$, respectively. If the treatment is perfect, patients will recover at the rate σ and return to the susceptible state. This is possible when the period of immunity is very short. Here, we neglect the effect of immunity for simplicity. Suppose that treatment fails with a probability denoted by *f*, patients being under treated must change their status to either carriers with a fraction ϵ, or symptomatic infection with a fraction 1−ϵ. Finally, we assume that carriers may progress to develop symptoms at the rate ψ.

Given that the total population *N* is constant, a Continuous-Time Markov Chain (CTMC) associated with the model is defined as

$$X\left(t\right)=\left(S(t),C(t),I(t),T(t)\right),\;t\in \left[0,\infty \right),$$

such that the state space of each component is {0,1,…,N}. We define a change of X(t) in a sufficiently small time interval Δt as ΔX(t)=X(t+Δt)−X(t). By the Markov property, we are able to write the infinitesimal transition probabilities of the model as shown in Table 1.

### 2.2. Extinction Threshold

We used a multi-type Galton–Watson branching process and a generating function technique to estimate the time to extinction during the early phase of an epidemic. The branching process is usually constructed by determining the offspring distributions derived from the transition rates in the infinitesimal transition probabilities of the continuous-time model. Near the disease-free equilibrium, the process assumes that each individual of the same infectious type and generation independently produces a random number of offspring, following a common distribution, and this production is also independent across generations [44,45]. Using a continuous-time branching process, the total number of individuals of each infectious type in each generation is estimated as the sum of all offspring, including their parents (i.e., the offspring of the previous generation) who remain of the same type [46].

Time to extinction in a branching process can be calculated based on an extinction threshold. Although the basic reproduction number has a qualitatively universal property, the exact extinction threshold in a stochastic model may differ. Following the methods of previous studies [47,48], we derive the probability of disease extinction, $P_{ext}$ which is used to characterize the stochastic threshold as follows.

The offspring probability generating functions (PGFs) are defined as

$$f_{i}\left(u_{1},\ldots ,u_{n}\right)=\sum_{k_{n}}\ldots \sum_{k_{1}}P_{i}\left(k_{1},\ldots ,k_{n}\right)u_{1}^{k_{1}}\ldots u_{n}^{k_{n}},$$

where $P_{i}$ is the probability that an infected individual of type *i* causes $k_{j}$ members of type *j* in the next generation. Using the transition probabilities in Table 1, where i=1 denotes carriers, i=2 denotes symptomatic infection, and i=3 denotes the under-treatment state, we have

(1) $$\begin{array}{cc} f_{1}\left(u_{1},u_{2},u_{3}\right) & =\frac{\left(1-\alpha \right)\beta N\eta u_{1}^{2}+\alpha \beta N\eta u_{1}u_{2}+\psi u_{2}+\theta_{c}u_{3}+\gamma \xi }{\beta N\eta +\psi +\theta_{c}+\gamma \xi },\\ f_{2}\left(u_{1},u_{2},u_{3}\right) & =\frac{\left(1-\alpha \right)\beta Nu_{1}u_{2}+\alpha \beta Nu_{2}^{2}+\theta_{i}u_{3}+\gamma }{\beta N+\theta_{i}+\gamma },\\ f_{3}\left(u_{1},u_{2},u_{3}\right) & =\epsilon fu_{1}+\left(1-\epsilon \right)fu_{2}+1-f. \end{array}$$

The expectation matrix $M=[m_{ij}]$, where $m_{ij}=\partial f_{i}/\partial u_{j}$ defined by the expected number of offspring of type *i* produced by a member of type *j*, can be calculated. The spectral radius of *M*, denoted by ρ(M) determines the extinction threshold. Specifically, if ρ(M)<1, then $P_{ext}=1$; but if ρ(M)>1, then $P_{ext}<1$. In the latter case, there exists a unique fixed point of the offspring PGFs (1), $(q_{1},q_{2},q_{3})\neq 1$, such that

(2) $$\begin{array}{c} P_{ext}=q_{1}^{C_{0}}q_{2}^{I_{0}}q_{3}^{T_{0}}, \end{array}$$

where $C\left(0\right)=C_{0}$, $I\left(0\right)=I_{0}$, and $T\left(0\right)=T_{0}$.

### 2.3. Deterministic Approach

Since a compartment representing individuals under treatment can be considered a form of medical intervention, we define $R_{T}$ as the control reproduction number, which accounts for the effects of treatment and its potential failure. Using the next-generation method, we calculate $R_{T}$ based on the deterministic counterpart. This result is traditionally expressed as the sum of two components, as follows.

(3) $$R_{T}=R_{I}+R_{C},$$

where

$$R_{I}=\beta N\left[\frac{\alpha \left(\gamma \xi +\left(1-f\right)\theta_{c}\right)+\psi +\left(1-\epsilon \right)f\theta_{c}}{\left(\gamma \xi +\psi +\theta_{c}\right)\left(\left(1-f\right)\theta_{i}+\gamma \right)+\gamma \epsilon f\left(\xi \theta_{i}-\theta_{c}\right)}\right],$$

and

$$R_{C}=\eta \beta N\left[\frac{\left(1-\alpha \right)\left(\left(1-f\right)\theta_{i}+\gamma \right)+\epsilon f}{\left(\gamma \xi +\psi +\theta_{c}\right)\left(\left(1-f\right)\theta_{i}+\gamma \right)+\gamma \epsilon f\left(\xi \theta_{i}-\theta_{c}\right)}\right],$$

describe the relative transmission potential of symptomatic infections and carriers, respectively. In the absence of treatment, the model becomes an SICS model. Thus, the basic reproduction number is given by

$$R_{0}=\left(\frac{\beta N}{\gamma }\right)\left(\frac{\alpha \gamma \xi +\psi +(1-\alpha )\gamma \eta }{\gamma \xi +\psi }\right).$$

Notably, the impact of treatment failure can be qualitatively assessed by examining how parameter variations affect $R_{T}$. Since $R_{T}$ and ρ(M) share the same threshold, parameter variation may reveal a parametric threshold. Even if such a threshold exists under certain conditions, it has been shown that the corresponding value in the stochastic model is identical to the one derived by solving the equation $R_{T}=1$ [34].

### 2.4. Probability Density Function of Minor Outbreak Duration

In the branching process approximation, an epidemic that fizzles out represents a minor outbreak, which can occur in both subcritical and supercritical processes. Thus, the probability of extinction is referred to as the probability of a minor outbreak. To compute the probability density function (PDF) of the time to extinction, we applied the generating function technique [33].

Suppose that the transition probability over the time interval of length *t*, starting from a state X(0)=(s,c,i,k) is denoted by

$$P_{\left(c,i,k),(l,m,n\right)}=P\left(\left(C(t),I(t),T(t)\right)=\left(l,m,n\right)|\left(C(0),I(0),T(0)\right)=\left(c,i,k\right)\right).$$

The corresponding backward Kolmogorov differential equation is

(4) $$\begin{array}{cc} \frac{d}{dt}P_{\left(c,i,k),(l,m,n\right)}= & \left(1-\alpha \right)\beta \left(i+\eta c\right)sP_{\left(c+1,i,k),(l,m,n\right)}+\alpha \beta \left(i+\eta c\right)sP_{\left(c,i+1,k),(l,m,n\right)}\\ \; & +\theta_{c}cP_{\left(c-1,i,k+1),(l,m,n\right)}+\psi cP_{\left(c-1,i+1,k),(l,m,n\right)}+\theta_{i}iP_{\left(c,i-1,k+1),(l,m,n\right)}\\ \; & +\sigma \left(1-f\right)kP_{\left(c,i,k-1),(l,m,n\right)}+\left(1-\epsilon \right)\sigma fkP_{\left(c,i+1,k-1),(l,m,n\right)}\\ \; & +\epsilon \sigma fkP_{\left(c+1,i,k-1),(l,m,n\right)}+\gamma iP_{\left(c,i-1,k),(l,m,n\right)}+\xi \gamma cP_{\left(c-1,i,k),(l,m,n\right)}\\ \; & -\left[\beta \left(i+\eta c\right)s+\sigma k+\left(\gamma +\theta_{i}\right)i+\left(\xi \gamma +\psi +\theta_{c}\right)c\right]P_{\left(c,i,k),(l,m,n\right)}. \end{array}$$

Given the initial condition (c,i,k), the probability generating function for (C,I,T) is defined as

$$G_{\left(c,i,k\right)}\left(z_{1},z_{2},z_{3},t\right)=\sum_{l,m,n}P_{\left(c,i,k),(l,m,n\right)}z_{1}^{l}z_{2}^{m}z_{3}^{n}.$$

By the assumptions of the branching process, we can estimate that

$$G_{\left(c,i,k\right)}\left(z_{1},z_{2},z_{3},t\right)={\left[G_{\left(1,0,0\right)}\right]}^{c}{\left[G_{\left(0,1,0\right)}\right]}^{i}{\left[G_{\left(0,0,1\right)}\right]}^{k}.$$

Thus, the PGF for any initial state can be computed from the PGFs corresponding to the three basis initial states. Using the backward Kolmogorov Equation (4) with initial conditions (1,0,0),(0,1,0), and (0,0,1), we obtain the following differential equations

(5) $$\begin{array}{cc} \frac{\partial }{\partial t}G_{\left(1,0,0\right)} & =\left(\beta N\eta +\psi +\theta_{c}+\gamma \xi \right)\left(f_{1}\left(G_{\left(1,0,0\right)},G_{\left(0,1,0\right)},G_{\left(0,0,1\right)}\right)-G_{\left(1,0,0\right)}\right),\\ \frac{\partial }{\partial t}G_{\left(0,1,0\right)} & =\left(\beta N+\gamma +\theta_{i}\right)\left(f_{2}\left(G_{\left(1,0,0\right)},G_{\left(0,1,0\right)},G_{\left(0,0,1\right)}\right)-G_{\left(0,1,0\right)}\right),\\ \frac{\partial }{\partial t}G_{\left(0,0,1\right)} & =\sigma \left(f_{3}\left(G_{\left(1,0,0\right)},G_{\left(0,1,0\right)},G_{\left(0,0,1\right)}\right)-G_{\left(0,0,1\right)}\right), \end{array}$$

where $f_{i}$, i=1,2,3 are the offspring PGFs in Equation (1). Since the solutions of Equation (5) do not depend explicitly on $z_{1},z_{2}$, and $z_{3}$, we solve them to obtain the probability of extinction at time *t*, given an initial condition at time 0. For example, $G_{\left(1,0,0\right)}\left(0,0,0,t\right)=P_{\left(1,0,0),(0,0,0\right)}\left(t\right)$. Once the solutions of system (5) are known, the PDF of time to extinction can be obtained by substituting them into the right-hand side of system (5).

### 2.5. Case Study and Scenarios

In this study, the effects of parameter variations on the duration of minor outbreaks are investigated using numerical simulations (see Appendix A). To this end, we employ parameter values from previous studies specifically describing bacterial infections caused by *Streptococcus pyogenes* (*S. pyogenes*) (see Table 2). Most parameter values were selected from ranges reported in the literature, while others were adjusted to fit our model.

For example, the overall estimated incidence of streptococcal carriers ranges between 2% and 25%, depending on the method of the study [18]. This suggests that the value of α lies within the interval [0.75,0.98]. In this study, we choose α=0.85. A longitudinal study of school-aged children reported a mean carriage duration of approximately 10 weeks [49]. To reflect this, we adjust the scaling factor ξ of the recovery rate for carriers to 0.189, corresponding to an average carriage duration of about 10.8 weeks. The exact rate at which carriers develop symptoms is not well-defined and may vary depending on individual and environmental factors; here, we set this rate to 0.01. The proportion of carriers identified after treatment also varies depending on the focus of the study. One report found that 15.6% of the children were still identified as *S. pyogenes* carriers after treatment [50]. We assume that this estimate is approximately representative of the general prevalence of carriers.

**Table 2:** Parameter values for modelling *S. pyogenes* transmission.

Symbol	Description	Value	Reference
βN	Transmission rate	0.45, 0.775	Assumed
α	Probability of symptomatic infection	0.85	[14,18]
γ	Untreated recovery rate of symptomatic individuals	0.07	[51,52]
ξ	Scaling factor of carrier recovery rate	0.189	[49]
$\theta_{c}$	Rate at which carriers undergo treatment	0.012	[34]
$\theta_{i}$	Rate at which symptomatic individuals undergo treatment	0.75	[34]
η	Scaling factor of carrier transmission rate	0.1	[34]
σ	Recovery rate under perfect treatment	0.5	[53]
ψ	Rate at which carriers develop symptoms	0.01	Assumed
ϵ	Fraction of treated patients who fail and become carriers	0.15	[50,54]

Although treatment failure can result from several factors, including incomplete adherence [19], antibiotic resistance [55] and reduced penicillin efficacy [56], pharyngeal carriage plays a key role in both the cause and the effect [17]. These salient features are captured by the model parameter *f*, which reflects the overall effectiveness of treatment, and the parameter ϵ, which represents the fraction of individuals under treatment who fail to recover and subsequently become carriers. This aligns with the observation that if the bacteria are not eradicated by a course of antimicrobial therapy, the pharynx may remain colonized with *S. pyogenes* without causing symptoms [57]. In addition, streptococcal carriers may receive unnecessary antibiotic therapy when presenting with a viral illness while testing positive for *S. pyogenes* on throat culture.

A systematic review study [58] reveals that the failure rate of penicillin treatment is relatively high, ranging from 14% to 40% for acute infections and from 30% to 87% for recurrent infections, compared with other drugs, which have failure rates of 0% to 14% for acute infections and 0% to 60% for recurrent infections. The effect of altering the parameter *f* will be examined under the conditions that

(i) a parametric threshold exists and
(ii) $R_{T}>1$ for all values of *f*.

These two scenarios allow for a comparison of minor outbreak durations between subcritical and supercritical processes, including inter-scenario comparisons under the supercritical condition. For this analysis, we set β=0.00045 in the first scenario and β=0.000775 in the second.

We note that the criteria for adjusting parameter values used as a baseline set are (see also Appendix A):

1. The values are selected to satisfy the existence condition for the parametric threshold. Although, the critical value can be computed from equation $R_{T}\left(f\right)=1$, the parametric threshold may not always exist [34].
2. The values are selected to satisfy the possible range of estimated basic reproduction number. Since the estimation of basic reproduction number for *S. pyogenes* in literature is rare, we use data of scarlet fever and investigated spatiotemporal spreading patterns of the disease with certain time lags in Hong Kong, Macau, and Guangdong in 2011. The estimated $R_{t}$ is between 0.6 and 2.0 [59].
3. The values are selected to satisfy the possible range of failure rate. Since the range is wide, the criterion to select parameter values is that the critical treatment failure probability should be intermediate so that we can investigate both the subcritical and supercritical regimes in the first scenario.

## 3. Results

### 3.1. Influence of Treatment Failure Probability on PDF Patterns

In the first scenario, we find that the extinction threshold as a function of the parameter *f*, occurs at $f_{c}=0.37$. This can be verified by observing that $R_{T}$ increases with *f*, and that $R_{T}=0.68$ when f=0. Thus, the epidemic almost surely fades out if $f<f_{c}$, but escapes extinction with probability $1-P_{ext}$ if $f>f_{c}$. The probability of a minor outbreak can be estimated only in the latter condition.

Conversely, in the second scenario, we find that $R_{T}=1.17$ when f=0, which is sufficient for the process to remain supercritical for all *f*. Hence, $P_{ext}$ can be estimated for every f∈[0,1).

It is worth noting that in the extreme case where f=1, the system may perform worse than under the no-treatment condition. Based on the model assumptions, if no individuals recover through therapy, then the average total infectious period would increase. Moreover, since 100% treatment failure is unrealistic, we exclude this case from our analysis. In addition, the initial condition in which a single individual is under treatment is also excluded.

**Figure 2:**
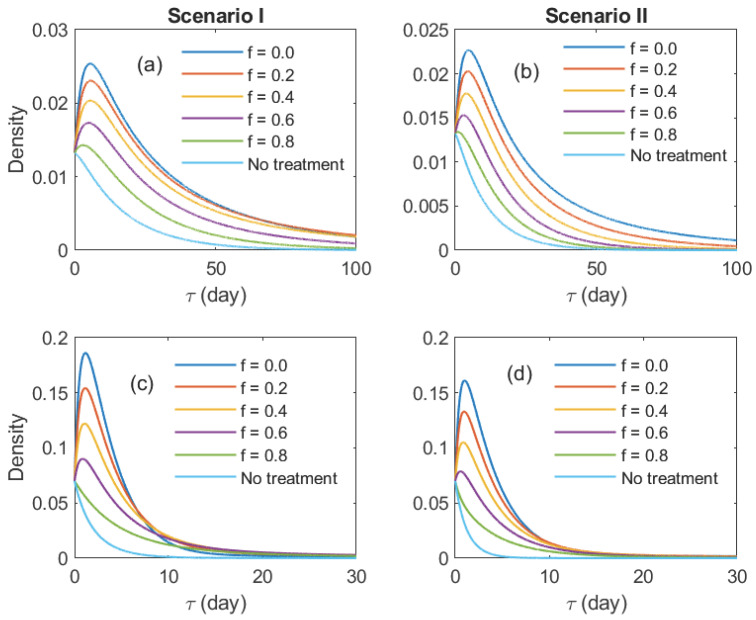
Probability density functions (PDFs) of minor outbreak durations computed from the probability generating function (PGF) approximations. The left column corresponds to a parameter set in which the *f*-induced extinction threshold exists, while the right column corresponds to a regime where $R_{T}>1$ for all *f*. From top to bottom, rows show PDFs for the initial conditions $\left(C_{0},I_{0},T_{0}\right)=\left(1,0,0\right)$ in (**a**,**b**), and $\left(C_{0},I_{0},T_{0}\right)=\left(0,1,0\right)$ in (**c**,**d**).

Figure 2 shows the PDFs of minor outbreak duration (τ) for various values of *f* and initial conditions. The PDFs were computed by solving system (5) numerically. When the system starts with a single untreated infectious individual (or carrier), the distributions exhibit a right-skewed, unimodal shape at low to intermediate values of *f*. Otherwise, the PDFs resemble an exponential-like decay. From a public health perspective, this indicates that most control efforts or preparedness should be focused on short-term spread while the presence of prolonged spread is expected to be of low likelihood. In both scenarios, the peak of the PDFs decreases as *f* increases. Notably, when the initial condition involves a carrier, both a decreasing peak and a thinner right tail are clearly observed, suggesting a reduced likelihood of prolonged outbreaks as the probability of treatment failure increases. This transition indicates a loss of epidemic control; higher failure rates do not just prolong the disease, they make the duration highly unpredictable and increase the risk of persistent, long-term transmission chains. The PDFs under the no-treatment regime show the lowest overall values compared to all other *f* levels. The overall shapes of the PDFs are similar across the two scenarios, except that the peaks in the second scenario are slightly lower at small to intermediate values of *f*.

### 3.2. Probability of Prolonged Persistence and Tail Risk

Solutions to Equation (5) yield the conditional probability distributions of the time to extinction–that is, the probability that the epidemic fades out by time *t*, given an initial condition. As time approaches infinity, these probabilities converge to the probability of a minor outbreak, $P_{ext}$, if it exists. Consequently, the probability of a major outbreak is simultaneously determined as $1-P_{ext}$. We now consider the threshold at which a major epidemic may occur under the duration-based metric [32]. This threshold is defined by the possible or maximum duration of a minor outbreak. In practice, it is useful to apply the duration criterion in combination with other thresholds, such as the availability of treatment and control resources.

**Figure 3:**
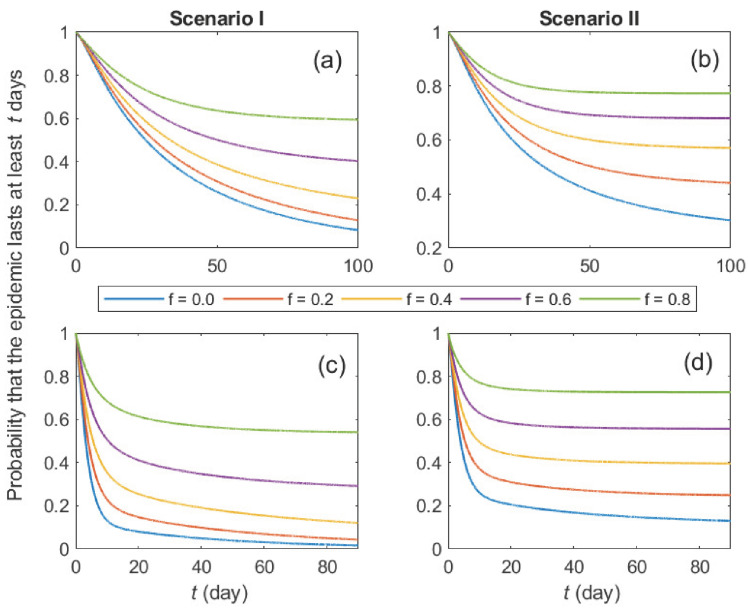
The probability that the epidemic survives longer than time *t* is illustrated for several levels of treatment failure. The probabilities converge to the probability of a major outbreak as time approaches infinity. The separation time between minor and major outbreak durations can be determined as a threshold according to the criterion of the control strategy. Subfigures (**a**,**b**) are for the initial conditions $\left(C_{0},I_{0},T_{0}\right)=\left(1,0,0\right)$. Subfigures (**c**,**d**) are for the initial conditions $\left(C_{0},I_{0},T_{0}\right)=\left(0,1,0\right)$.

As the duration threshold can be arbitrarily adjusted when used alongside other thresholds, we compute the probability that a duration threshold can be defined for various values of the treatment failure probability (Figure 3). Nevertheless, factors influencing outbreak duration, such as transmission settings, population immunity, and public health interventions, introduce uncertainty in defining such a threshold. For example, the duration of minor outbreaks caused by *S. pyogenes* transmission has been reported to range from a few months (e.g., clusters in hospitals and care facilities) to as long as a year (e.g., in nursing homes) [53,60,61].

**Figure 4:**
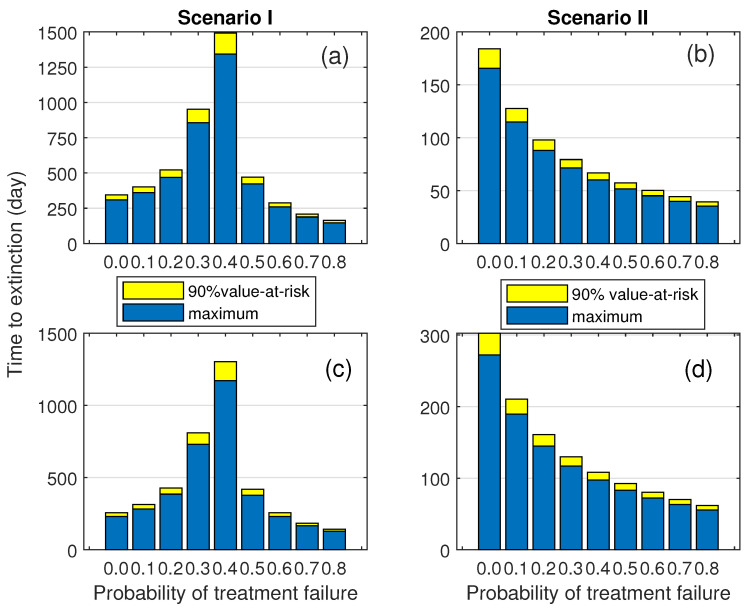
The effects of treatment failure on the approximated maximum duration of minor outbreak calculated from the PGFs and corresponding 90th percentiles. Subfigures (**a**,**b**) are for the initial conditions $\left(C_{0},I_{0},T_{0}\right)=\left(1,0,0\right)$. Subfigures (**c**,**d**) are for the initial conditions $\left(C_{0},I_{0},T_{0}\right)\;=\;\left(0,1,0\right)$.

Based on the parameter values in Table 2, we used the PGFs to estimate the maximum and the 90th percentile of the minor outbreak duration (Figure 4). These estimates reflect the possible range of minor outbreak durations and quantify the extent of that possible range within 90% confidence level. The widening of the yellow band (representing the 90th percentile interval) as the treatment failure rate approaches the critical threshold illustrates a significant increase in stochastic uncertainty. Near the threshold, the outbreak duration becomes highly unpredictable, with some chains fading out quickly while others persist for extended periods due to critical slowing down.

In the first scenario, a pronounced peak in the estimated quantities occurs at a vicinity of the critical value of *f*. As *f* exceeds through this critical value, the process becomes supercritical, and both the estimated maximum and the 90th percentile steadily decrease in both scenarios. When the maximum duration is prolonged, data points tend to cluster toward the lower end of the scale, with a few extreme values extending to the higher end. This pattern corresponds to the PDFs observed in the previous section. Interestingly, the observed exponential decay of durations in the supercritical regime does not imply improved control. Instead, it indicates a regime shift where the majority of transmission chains successfully transition into major outbreaks. For public health policy, this suggests that beyond the critical failure rate, clinical treatment alone fails as a containment strategy, and the focus must shift entirely to population-level interventions.

### 3.3. Statistics of Simulated Data

Measuring the central tendency and variability of the estimated PDFs in this context is a challenging task due to the presence of outliers. Analytical expressions for the moments of the minor outbreak duration are only available when the model can be approximately reduced to a birth-death process [33]. In this study, we simulated stochastic trajectories using the Gillespie algorithm to capture random fadeout dynamics. The means and medians of the simulated datasets were compared across a range of treatment failure probabilities.

**Figure 5:**
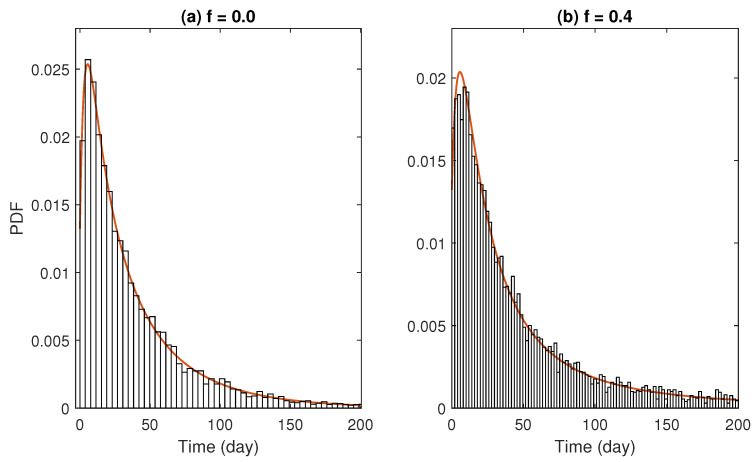
Examples of PDFs when f=0.0 (**a**) and f=0.4 (**b**) created from numerical simulations (bars) compared with analytical results computed from PGF approximations (solid lines). Both subplots were created using parameters from the first scenario and the initial condition $\left(C_{0},I_{0},T_{0}\right)\;=\;\left(1,0,0\right)$.

Figure 5 shows that the PDFs of the simulated data agree well with the analytical results derived in the previous section. This consistency is verified for every instance of the calculations. In the simulations, the sample size is set to $10^{5}$. For each realization, the time to extinction, denoted by τ, is recorded, and the corresponding probability is estimated by counting the instances when the absorbing state is reached. The absolute error of the estimated probability is computed using Equation (2). The time range used in each simulation is adaptively adjusted to minimize this error. Although this procedure is necessary for computational efficiency, it introduces some numerical instability.

A long simulation time window may produce a significant number of outliers, which can affect the precise estimation of the mean. Due to the right-skewed distribution, potential outliers on the right side are identified as values exceeding the upper bound defined by the third quartile plus 1.5×IQR, where the interquartile range (IQR) represents the spread of the middle 50% of the data. We note that only the situations where the system starts with a single actively infected individual are focused. Therefore, the simulations are conducted under two initial conditions. The results are summarized in Table A1.

**Figure 6:**
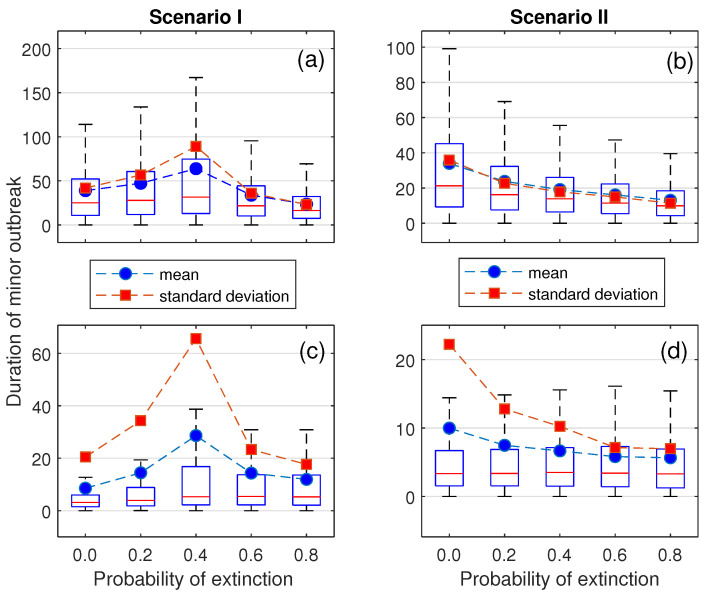
Comparison of simulated minor outbreak durations versus treatment failure probability for different scenarios and initial conditions. Subfigures (**a**,**b**) are for the initial conditions $\left(C_{0},I_{0},T_{0}\right)\;=\;\left(1,0,0\right)$. Subfigures (**c**,**d**) are for the initial conditions $\left(C_{0},I_{0},T_{0}\right)\;=\;\left(0,1,0\right)$. Central tendency and variability of each distribution are shown.

Box-and-whisker plots of the simulated datasets against stepwise values of *f* are shown for different scenarios and initial conditions in Figure 6. The estimated means and medians are clearly distinct in terms of scale and changing pattern across scenarios and initial conditions. Apart from the outliers effect, the observed gap between the mean and median may suggest the accumulation of longer outbreak durations in the right tail, indicating a heavier or denser tail structure.

In the first scenario, the qualitative change in the mean duration of minor outbreaks as *f* increases follows the analytical results derived in the SIR, SIS, and SEIR models, where $R_{0}$ is treated as a varying parameter. However, the change in medians—especially when the system starts with a single symptomatic individual—differs slightly: the peak appears to be delayed by one step beyond the threshold value. As *f* increases, the estimated means exhibit more substantial fluctuations than the medians. Moreover, the divergence between the mean and median becomes particularly evident when *f* approaches the critical threshold.

In the second scenario, when a single carrier is introduced, both the means and medians exhibit a downward trend as *f* increases. This pattern is consistent with that observed in the first scenario, where *f* continually shifts away from its threshold value. When a single symptomatic infectious individual is introduced, the medians across different values of *f* remain relatively stable. Additionally, the divergence between the means and medians decreases as *f* increases.

**Figure 7:**
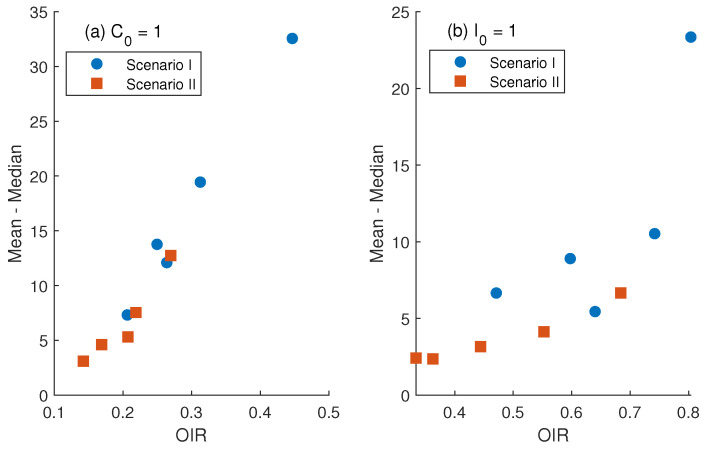
Increasing gaps between means and medians as the effect of outliers increases. These gaps are calculated for each simulated minor outbreak duration. Left panel (**a**) is for the initial conditions $\left(C_{0},I_{0},T_{0}\right)=\left(1,0,0\right)$, and right panel (**b**) is for the initial conditions $\left(C_{0},I_{0},T_{0}\right)=\left(0,1,0\right)$. Each subfigure shows the results from two scenarios with different initial conditions.

Unusual long outbreak durations are rare but may be treated as outliers. To assess how such outliers distort the statistics of the simulated data, especially the means, we compute the Outlier Influence Ratio (OIR), defined as the percentage of the total sum contributed by data points classified as outliers. The difference between the means and medians is plotted against the OIR (see Figure 7). The results show that the gap between the mean and median increases approximately in direct proportion to the OIR. While the median remains relatively stable, the increasing gap highlights the sensitivity of the mean to changes in the tail. In our case, the condition starting with a single carrier appears more sensitive to the influence of outliers on the mean, as indicated by the steeper slope of the approximated linear relationship between the mean–median gap and the Outlier Influence Ratio (OIR).

### 3.4. Effects of Initial Conditions

The probability of a minor outbreak appears to be inversely related to both the probability of treatment failure and the number of initial infections. A low probability virtually redirects attention to major outbreak scenarios. To assess how the means and medians of outbreak duration change with an increasing number of initially infected individuals, we restrict the simulations to cases where the epidemic starts with three initially infected individuals and where the probability of a minor outbreak exceeds 80%.

Since the second scenario focuses on parameter sets that support the emergence of major outbreaks, we estimated the means and medians of the simulated data only for the first scenario. The results, shown in Table 3, indicate the percentage change in the medians, means, and Outlier Influence Ratio (OIR) relative to the case where a single infected individual is introduced. Unlike the previous analysis, the range of *f* is limited to values near the threshold. Beyond this range, the probability of a minor outbreak drops below 30% for the initial condition $C_{0}=3$, and below 40% for $I_{0}=3$.

Increasing the number of initially infected individuals from one to three does not significantly affect the shape of the PDFs of the time to extinction. However, the median outbreak duration increases by a factor of 1.2 to 3.7, and the mean increases by a factor of 0.8 to 1.5. Overall, the influence of outliers decreases. Both the means and medians increase with *f* up to the vicinity of the threshold, indicating that the behavior of the central tendency remains consistent under small changes in the initial condition.

According to the suggested period of minor outbreaks [53,60,61] the results from Figure 6a,b give the range of an epidemic period ranging from about 2 weeks to 2 months. A single symptomatic infection may not be sufficient to generate a sufficiently long outbreak duration. This implies that starting with a single carrier or with several symptomatic infections is more likely to generate a minor outbreak period that matches the real data.

### 3.5. Sensitivity Analysis

To identify the most influential parameter and to inform the degree of uncertainty for patterns under an increase in treatment failure probability, we calculated local sensitivity indices for the estimated mean duration of a minor outbreak denoted by $\hat{\tau }$ with respect to the model parameters. A series of tests are performed on the baseline parameter values in Table 2. Suppose that *p* is an input parameter. Due to the lack of an explicit mean-duration formula, the normalized forward sensitivity index of the output is approximated as

(6) $$\frac{p}{\hat{\tau }}\frac{\partial \hat{\tau }}{\partial p}\approx \left(\frac{p}{\hat{\tau }}\right)\frac{\left(\hat{\tau }\left(p+\Delta p\right)-\hat{\tau }\right)}{\Delta p}$$

**Figure 8:**
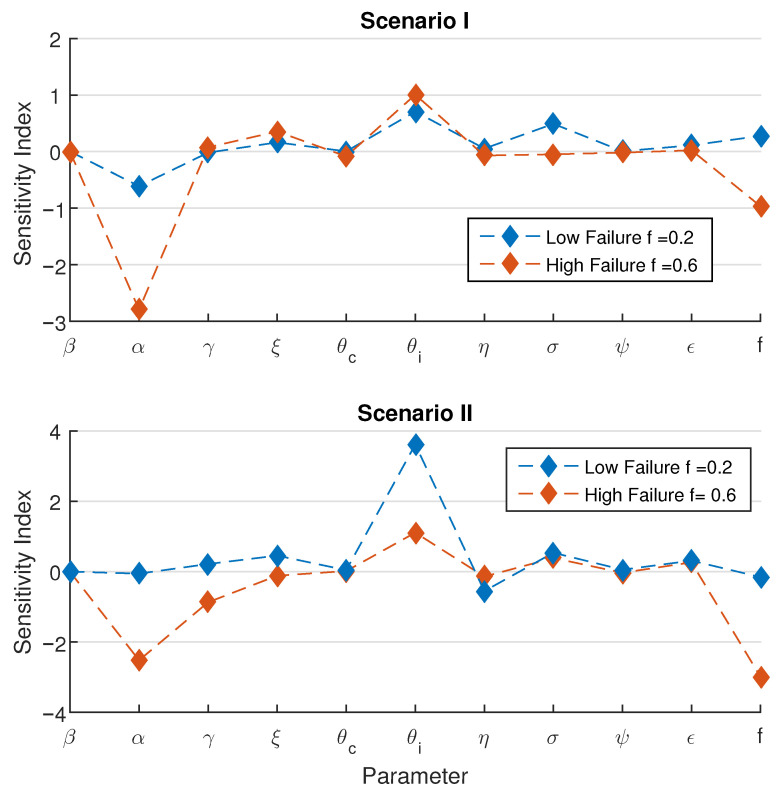
Sensitivity of model parameters on the estimated mean duration of minor outbreak. Using the baseline parameters in Table 2, top panel represents the first scenario Nβ=0.45, and the below panel represents the second scenario Nβ=0.775. For both figures, we use the same initial conditions $\left(C_{0},I_{0},T_{0}\right)=\left(1,0,0\right)$.

For the 11 input parameters per scenario to be tested, changing the value of *p* is taken as a 1% increase in the positive direction. For each set of parameter values, two further specific situations are considered: low failure using f=0.2, and high failure using f=0.6. The results are shown in Figure 8.

In the first scenario, the two most sensitive parameters are $\theta_{i}$, the treatment rate of symptomatic patients, and α, the fraction of incidence that becomes symptomatic infection. The magnitude of both sensitivity indices is relatively high in the high failure condition. An increase in α implies that the chance of new carriers is lower, hence reducing the fraction of long epidemic durations, resulting in a reduce mean duration. On the other hand, if the flow rate from symptomatic infection to treatment is accrued, then the chance of obtaining a new member of the treatment class increases, subsequently increasing the chance of new carriers. The presence of carriers has a positive effect on prolonging the time to extinction.

In the second scenario, we find that α becomes less sensitive compared to the parameter $\theta_{i}$, under the low failure condition. Yet, these two parameters become predominant again under the high failure condition. Surprisingly, the sensitivity of the treatment failure probability, *f* is also comparable in the high failure condition. It is noted that the sign of the sensitivity index of *f* is consistent with the observed patterns for both scenarios.

### 3.6. Marginal Effect of Transmission During Treatment

In this section, we examine the robustness of the unimodal relationship between mean minor outbreak duration and treatment failure probability against the marginal influence of transmission by treated individuals. If the model assumption that individuals in the treatment state are completely non-infectious is relaxed, the force of infection becomes

(7) β(I+ηC+νT).

Although patients may still have a certain degree of infectivity during treatment, they typically exhibit lower infectivity compared to untreated individuals at the population level. We now focus on two levels, that is, 5% and 10% of the transmission coefficient β.

**Figure 9:**
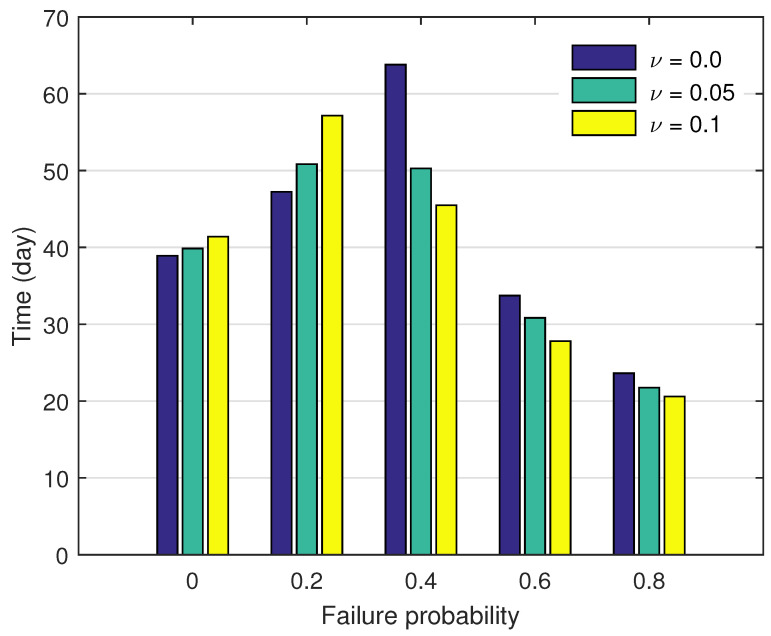
Mean minor outbreak duration as a function of the treatment failure under different levels of transmission during treatment. The baseline case assumes zero transmissibility in the treatment class (ν=0). Two additional scenarios allow reduced transmissibility during treatment, with ν=0.05 and ν=0.1, respectively. Results are obtained from stochastic simulations for each parameter set together with the first scenario Nβ=0.45 and the initial conditions $\left(C_{0},I_{0},T_{0}\right)=\left(1,0,0\right)$. The qualitative relationship between treatment failure rate and mean minor outbreak duration remains consistent across all scenarios.

Figure 9 shows the simulation results from the modified force of infection. The pattern for ν=0 coincides with the pattern in Figure 6a. It is seen that, in the subcritical regime, the mean outbreak duration increases as the transmission potential of the under-treatment state increases. For the five-percent case, the outbreak duration increases by 2.43% and by 7.62% when using f=0 and f=0.2, respectively. In the supercritical regime, the reverse effect is observed. The outbreak duration decreases by 21.17%, 8.56% and 7.89% when using f=0.4,0.6 and 0.8, respectively. For the ten-percent case, the outbreak duration increases by 6.41% and by 21.01% when using f=0 and f=0.2, in the subcritical regime. Whereas it decreases by 28.69%, 17.58% and 12.79% when using f=0.4,0.6 and 0.8, respectively, in the supercritical regime.

Overall, the unimodal pattern remains qualitatively unchanged under the introduction of marginal transmission during treatment. We note that the observed scale shifts are due to a decrease in the critical value (threshold) for the treatment failure probability. Under the main assumption, we have $f_{c}=0.37$. For ν=0.05 and 0.1, we have $f_{c}=0.32$ and 0.27, respectively. Since the peak of the unimodal curve occurs in the vicinity of this threshold, the horizontal shift in $f_{c}$ naturally induces the observed displacement in scale.

In the supercritical regime, including the scenario when a threshold does not exist, it can be deduced that the inverse relationship between the mean outbreak duration and the treatment failure probability holds under the same marginal effect. The reduction in scale may be observed since an increase in the control reproduction number which reduces the probability of extinction and consequently shortens the expected duration of minor outbreaks.

## 4. Discussion

We have demonstrated how the effectiveness of treatment, in synergy with asymptomatic transmission, influences the duration of minor outbreaks for a specific pathogen. *Streptococcus pyogenes* transmission was used as a case study, given the prominent role of asymptomatic carriers in bacteriological treatment failure. As the probability of treatment failure increases, the expected duration of minor outbreaks exhibits a pattern consistent with previous studies in which the basic reproduction number was treated as the independent variable. This pattern is characterized by a unimodal shape, with a peak occurring near the threshold. However, due to differences in model structure and key driving mechanisms, the observed similarity is constrained to parameter conditions that permit the existence of a well-defined threshold.

**Figure 10:**
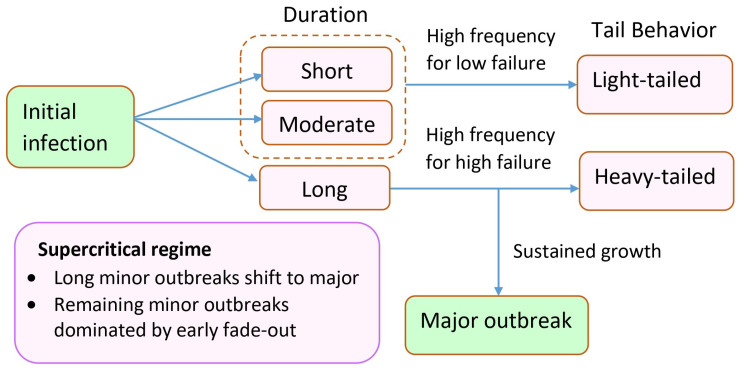
Schematic illustration of how increasing treatment failure reshapes extinction pathways in the supercritical regime.

Recall that the model in this study incorporates both asymptomatic transmission and recurrent infections through treatment and recovery. The interaction between stochastic fade-out and transmission potential can help explain the emergence of the unimodal pattern. At very low values of *f*, early fade-out is almost guaranteed and typically occurs over a short duration. Since *f* reduces the recovery rate from the under-treatment state, increasing its value enhances transmission potential by allowing infected individuals to remain in the system longer. This excess transmissibility can lead to prolonged outbreak durations. The mean and variability of these durations reach their peak as *f* approaches the threshold value. However, once *f* becomes large enough to push the system into a supercritical regime, the opposite effect is observed. As *f* increases, the control reproduction number increases so that the number of infections continually grows. The turning point is that although the probability of major outbreak increases, the remaining paths remain small outbreak with moderate and short duration due to the stochastic fade-out. Under the condition of minor outbreak, the sustained growth that becomes a major outbreak is uncounted. This leads to the distribution change in tail behavior, from heavy-tailed to light-tailed if the large fraction of paths with long extinction time is neglected. Thus, although transmissibility increases, the chance that an infection persists for a long time before dying out becomes increasingly rare. This pattern is presented in Figure 4. We have seen a similar pattern of mean duration in Figure 6. The summarized diagram is shown in Figure 10.

Although an exact analytical relationship between the expected duration of minor outbreaks and the probability of treatment failure is not available, simulation results reveal several important features—particularly when the treatment failure probability exceeds the threshold. Importantly, the expected durations of minor outbreaks in the two scenarios differ significantly, even when the control reproduction number and the probability of extinction are nearly identical. This contrast is especially evident when the treatment failure probability is 60% in the first scenario and 20% in the second, both yielding a control reproduction number of approximately 1.42. When starting with a single carrier, the estimated mean duration is 33.73 days in the first scenario and 16.24 days in the second. When starting with a single symptomatic individual, the corresponding means are 14.32 days and 7.48 days, respectively. These results suggest that the expected duration of minor outbreaks is not uniquely determined by the control reproduction number or the basic reproduction number alone. Therefore, in a supercritical regime, the expected duration should also be characterized based on whether a parametric threshold exists and whether it plays a key role in shaping the transmission dynamics.

We note that the observed unimodal pattern of expected duration may be objective since our model is not in the same class as the SIRS or SEIR models presented in previous work [33]. In a generalized perspective, we hypothesize that the pattern of the relationship between the expected duration and other model parameters is similar under the range that contains a threshold. This indicates that the parametric threshold may act as a singularity in the formula for the expected duration of a minor outbreak, similar to the case of the basic reproduction number. Moreover, the form of analytical formulas, if they exist, should be characterized according to the existence conditions of the parametric threshold.

Since the estimated probability density functions (PDFs) of minor outbreak durations are right-skewed, the influence of outliers should be carefully evaluated and, if necessary, mitigated. A high outlier influence ratio may lead to overestimation of the mean. Trimmed statistics, such as 5% truncation, can reduce the impact of extreme values and stabilize summary measures. However, trimming may also obscure important rare events—specifically, unusually long minor outbreaks. To balance statistical robustness with the need to capture meaningful outcomes, more stable measures of central tendency, such as the median, were reported along with interquartile range (IQR). The results show that truncation affects the mean, but the median and IQR remain relatively stable.

Depending on the type of initially infected individual, the central tendency of minor outbreak durations as a function of treatment failure probability displays different scales. On average, infections tend to persist longer when a single carrier is introduced compared to when a symptomatic individual initiates the outbreak. This is primarily because the recovery rate of carriers is much lower than that of symptomatic individuals. Parameter values derived from observational data indicate that the duration of the carrier state is approximately 5.3 times longer than the infectious period of symptomatic cases. When the system starts with a carrier, stochastic fade-out typically occurs after a moderate duration. Since carriers undergo treatment at a relatively low rate, the system may spend a substantial portion of time in the carrier state before dying out. On the other hand, when the outbreak starts with a symptomatic individual, the chance of transitioning to the carrier state is low, as only a small proportion of under-treatment cases flow into the carrier compartment. As a result, the system tends to be dominated by symptomatic infections. In this case, most outbreaks either fade out quickly or persist for a relatively long period. Consequently, the outlier influence ratio (OIR) is higher when the system starts with a symptomatic individual than with a carrier.

Our study presents some limitations. In addition to having limited high-quality data on minor outbreaks, our understanding may be restricted by the uncertainty of carrier transmission potential and the assumption of an exponentially distributed infectious period. For *S. pyogenes*, the carrier state is not well understood regarding both its pathogenesis and transmissibility [18]. The infectious period of carriers is also poorly defined. Even though the carriage state may last for several weeks, the most contagious period is about the first couple of weeks and then decreases over time [62]. These factors create gaps that prevent precise estimation of model parameters. Thus, additional model development is still required to gain a better insight into the role of carriers on both treatment failure and minor outbreak-specific dynamics.

However, the structure of the proposed model allows for several extensions and modifications to be applied to many infectious diseases involving carriers or asymptomatic infection. For instance, to adapt the model for COVID-19 infections, latent and recovery stages can be added. The stage under treatment can be changed to hospitalization or quarantine depending on the purpose of the study. Our model can also be adjusted for Tuberculosis transmission [63] and Meningococcal Meningitis [26,43]. Furthermore, since the distribution of infectious periods significantly impacts outbreak duration [64], using alternative probability distributions for carrier infectious period can better represent the uncertainty inherent in minor outbreak dynamics [65,66].

Our result indicates the importance of bacteriological failure in *S. pyogenes*, which has clinical implications. Carriers of *S. pyogenes* may receive treatment unnecessarily due to viral infections that present common symptoms [67,68]. This is a type of treatment failure presented in our model. Treatment failure can also lead to persistent carriage of *S. pyogenes*, and such asymptomatic carriers can still transmit the bacteria to others or progress to symptomatic infection, leading to potential outbreaks. Although the epidemic may be minor, a long duration may have significant consequences, such as the outbreak within healthcare facilities [53,60,61]. In addition, there were several instances of household transmission clusters (H5N1 and COVID-19) that later became pandemic [29]. Understanding and predicting the relationship between treatment failure and minor outbreak duration may guide the outbreak management by informing resource allocation, intervention timing, and supporting evidence-based policy decisions.

Future studies should focus on improving the methodology for deriving the central tendency of time to extinction using both analytical and simulation frameworks. To move beyond simple models and better capture uncertainty, the sensitivity of the derived duration to model structural extensions should be investigated, which would allow for a focus on the impact of infectious period variation. The current model assumes homogeneous mixing, which provides a useful mean-field approximation. However, in real-world scenarios, contact heterogeneity or network structures can significantly influence outbreak dynamics. Specifically, in small populations, structured mixing may increase extinction probabilities due to localized saturation of susceptible individuals. While these factors might shift the exact numerical value of the parametric threshold, we expect our qualitative findings regarding the impact of treatment failure to remain robust across different mixing patterns. Furthermore, time to peak, final size, and the quasi-stationary distribution for the major epidemic should also be considered in combination with more complex models.

## 5. Conclusions

In our modelling framework, the results pose a challenge to redefining the expected minor outbreak duration as a function of the parameter affecting the infectious period instead of the traditional basic reproduction number. In our model, treatment acts as a critical clinical control measure by actively reducing the host’s infectiousness and shortening the duration of pathogen shedding. In contrast, treatment failure elevates the probability of secondary transmissions by prolonging the overall infectious period, resulting in extending the outbreak duration. The threshold of treatment failure identifies the maximum tolerable error for medical intervention. In the proximity of the threshold, an unusually long period pattern is underscored. These rare extreme events, characterized by prolonged transmission despite a low case count, imply a critical early warning signal of a potential major outbreak that was successfully contained through timely intervention.

We show that the pattern of the outbreak duration, when the threshold value of treatment failure probability exists, is different from when such a threshold does not exist. This provides a theoretical framework to distinguish between self-limiting small-scale outbreaks and early-stage major epidemics.

Improving the effectiveness of treatment alone may not be sufficient to break the chain of transmission or the lingering spread. This highlights a crucial biological reality. Clinical treatment only addresses the symptomatic fraction of the population since the asymptomatic carriers act as a hidden reservoir for the pathogen. While treatment targets identified cases, undetected shedding from asymptomatic individuals allows the chain of transmission to persist undetected by clinical screening. Proactive interventions such as efficient testing, identifying and isolating asymptomatic cases should be further focused on.

## Figures and Tables

**Table 1:** The infinitesimal transition probabilities of the continuous-time model.

Event	(ΔS,ΔC,ΔI,ΔT)	Transition Probability
Infection to *C*	(−1,1,0,0)	(1−α)β(I+ηC)S▵t+o(▵t)
Infection to *I*	(−1,0,1,0)	αβ(I+ηC)S▵t+o(▵t)
Flow from *C* to *T*	(0,−1,0,1)	$\theta_{c}C▵t+o\left(▵t\right)$
Flow from *C* to *I*	(0,−1,1,0)	ψC▵t+o(▵t)
Flow from *I* to *T*	(0,0,−1,1)	$\theta_{i}I▵t+o\left(▵t\right)$
Recover from *T* to *S*	(1,0,0,−1)	σ(1−f)T▵t+o(▵t)
Flow from *T* to *C*	(0,1,0,−1)	ϵσfT▵t+o(▵t)
Flow from *T* to *I*	(0,0,1,−1)	(1−ϵ)σfT▵t+o(▵t)
Recover from *I* to *S*	(1,0,−1,0)	γI▵t+o(▵t)
Recover from *C* to *S*	(1,−1,0,0)	ξγC▵t+o(▵t)

**Table 3:** The effect of small elevating initial conditions. Relative changes in means, medians and OIR are calculated only for cases where the probability of extinction is greater than 80%.

Scenario I						
**Initial Condition**	f	$P_{\mathrm{ext}}$	**Error (** $10^{-4}$ **)**	**Median (PC)**	**Mean (PC)**	**OIR (PC)**
$\left(C_{0},I_{0},T_{0}\right)=\left(3,0,0\right)$	0.0	1.00	0.00	139.80%	88.22%	−50.92%
	0.2	1.00	0.00	156.77%	95.24%	−48.85%
	0.4	0.83	7.65	173.90%	100.51%	−51.83%
$\left(C_{0},I_{0},T_{0}\right)=\left(0,3,0\right)$	0.0	1.00	0.00	121.55%	124.44%	4.81%
	0.2	1.00	0.00	185.65%	153.16%	−34.33%
	0.4	0.90	0.69	371.37%	140.19%	−41.62%

**Figure A1:**
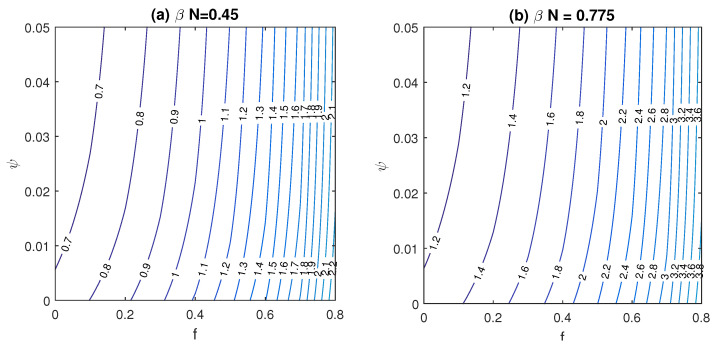
Contour plots of the control reproduction number in (3) as a function of *f* and ψ. Panel (**a**) is for the first scenario, and panel (**b**) is for the second scenario.

## Data Availability

The complete MATLAB codes and scripts used to generate all simulation results and figures are publicly available at: https://github.com/klotpat/carriers-epidemic-treatment-failure.git (accessed on 30 May 2025).

## Abbreviations

The following abbreviations are used in this manuscript:

AMR	Antimicrobial resistance
CTMC	Continuous-Time Markov Chain
SCIT	Susceptible-Carriers-Infectious-Treatment
PDF	Probability density function
PGF	Probability generating function
IQR	Interquartile range
OIR	Outlier Influence Ratio
PC	Percentage change

## Appendix A. Computational Procedure and Parameter Values Selection

### Appendix A.1. Path Simulation

We perform stochastic simulations of a continuous-time Markov chain epidemic model using the Gillespie algorithm. At any given time *t*, the total event rate λ is computed as the sum of all possible transition rates, including infection, progression, recovery, and treatment events. The waiting time to the next event is sampled from an exponential distribution with parameter λ. Conditional on the occurrence of an event, the specific transition is selected according to its relative contribution to the total event rate. The state vector is updated according to the net change of the selected event. Non-negativity of all compartment sizes is enforced at each event update. This procedure is repeated until either disease extinction occurs or a predefined maximum simulation time, $t_{max}$, is reached.

### Appendix A.2. Numerical Implementation

The initial values of S(0),I(0),C(0), and T(0) are set according to the scenario under the condition N=1000. Disease extinction is defined as the absorbing state I(t)=C(t)=T(t)=0. For each parameter set, 10,000 sample paths are generated. Simulations are run until either disease extinction occurs or $t_{max}$, is reached. For each realization, the extinction time is recorded. If extinction does not occur before $t_{max}$, the simulation is terminated and the final time is treated as right-censored. All algorithms are implemented in MATLAB R2018a. The simulations use MATLAB’s built-in pseudorandom number generator through the rand function, which generates independent samples from the continuous uniform distribution on (0,1). Parameter values are reported in the main text.

### Appendix A.3. Estimation of Extinction Probability

The extinction probability is estimated as the proportion of sample paths in which the disease dies out before $t_{max}$. This simulated extinction probability is compared with an analytically derived extinction probability obtained from a branching process approximation. The absolute error is used to assess the accuracy of the estimation. To ensure that the recorded duration is for minor outbreak, we run simulation several times for different value of $t_{max}$ until the absolute error is less than $10^{-3}$.

### Appendix A.4. Parameter Values Selection

In this study, the parameter ψ is introduced. To verify that the selected value used in this work is reasonable and meet the proposed criteria (see Section 2.5), we calculate the control reproduction number (3) for several values of *f* and ψ. Considering the empirical estimation of the reproduction number of *S. pyogenes* such that the values fall in the range [0.6,2.0] together with the systematic review study that gives the range of *f* as [14%,87%], we obtain the possible range of ψ as [0,0.05] for both scenarios (see Figure A1). Since the development from carriers to symptomatic infection is occasional and requires further supporting evidence, we set ψ=0.01 for this study. From the sensitivity analysis (see Section 3.5), we find that the variation of ψ in this range has a very low impact on the subsequent results.

## Appendix B. Supplementary Data

According to Figure 6, the supplementary information such as the control reproduction number ($R_{T}$), probability of extinction ($P_{ext}$), absolute errors, Interquartile range (IQR), and Outlier Influence Ratio (OIR) are shown in the following table.

**Table A1:** Supplementary information for the statistics of simulated data.

Scenario I						
**Initial Condition**	f	$R_{T}$	$P_{\mathrm{ext}}$	**Error (** $10^{-4}$ **)**	**IQR**	**OIR**
$\left(C_{0},I_{0},T_{0}\right)=\left(1,0,0\right)$	0.0	0.68	1.00	0.00	41.28	0.25
	0.2	0.82	1.00	0.00	48.88	0.31
	0.4	1.04	0.94	1.21	60.23	0.45
	0.6	1.42	0.64	2.16	34.10	0.26
	0.8	2.23	0.41	1.66	24.80	0.21
$\left(C_{0},I_{0},T_{0}\right)=\left(0,1,0\right)$	0.0	0.68	1.00	0.00	4.48	0.64
	0.2	0.82	1.00	0.00	7.05	0.74
	0.4	1.04	0.97	3.40	14.64	0.80
	0.6	1.42	0.73	0.78	11.48	0.60
	0.8	2.23	0.46	3.73	11.52	0.47
**Scenario II**						
**Initial Condition**	f	$R_{T}$	$P_{\mathrm{ext}}$	**Error (** $10^{-4}$ **)**	**IQR**	**OIR**
$\left(C_{0},I_{0},T_{0}\right)=\left(1,0,0\right)$	0.0	1.17	0.75	3.18	35.92	0.27
	0.2	1.42	0.57	3.76	24.66	0.22
	0.4	1.80	0.43	3.78	19.68	0.21
	0.6	2.45	0.32	0.09	16.82	0.17
	0.8	3.84	0.23	5.79	14.10	0.14
$\left(C_{0},I_{0},T_{0}\right)=\left(0,1,0\right)$	0.0	1.17	0.89	1.38	5.15	0.68
	0.2	1.42	0.76	1.06	5.32	0.55
	0.4	1.80	0.61	2.54	5.63	0.44
	0.6	2.45	0.44	1.32	5.90	0.33
	0.8	3.84	0.27	3.82	5.70	0.36
